# How Do Gangliosides Regulate RTKs Signaling?

**DOI:** 10.3390/cells2040751

**Published:** 2013-12-06

**Authors:** Sylvain Julien, Marie Bobowski, Agata Steenackers, Xuefen Le Bourhis, Philippe Delannoy

**Affiliations:** 1 Structural and Functional Glycobiology Unit, UMR CNRS 8576, University of Sciences and Technologies of Lille, 59655 Villeneuve d’Ascq, France; E-Mails: sylvain.julien@univ-lille1.fr (S.J.); marie.bobowski@hotmail.fr (M.B.); agata.steenackers@hotmail.fr (A.S.); 2 INSERM U908, University of Sciences and Technologies of Lille, 59655 Villeneuve d’Ascq, France; E-Mail: xuefen.lebourhis@univ-lille1.fr

**Keywords:** receptor tyrosine kinase, gangliosides, glycosylation, cell signaling, glycolpid-enriched microdomains

## Abstract

Gangliosides, the glycosphingolipids carrying one or several sialic acid residues, are located on the outer leaflet of the plasma membrane in glycolipid-enriched microdomains, where they interact with molecules of signal transduction pathways including receptors tyrosine kinases (RTKs). The role of gangliosides in the regulation of signal transduction has been reported in many cases and in a large number of cell types. In this review, we summarize the current knowledge on the biosynthesis of gangliosides and the mechanism by which they regulate RTKs signaling.

## 1. Introduction

**Figure 1:**
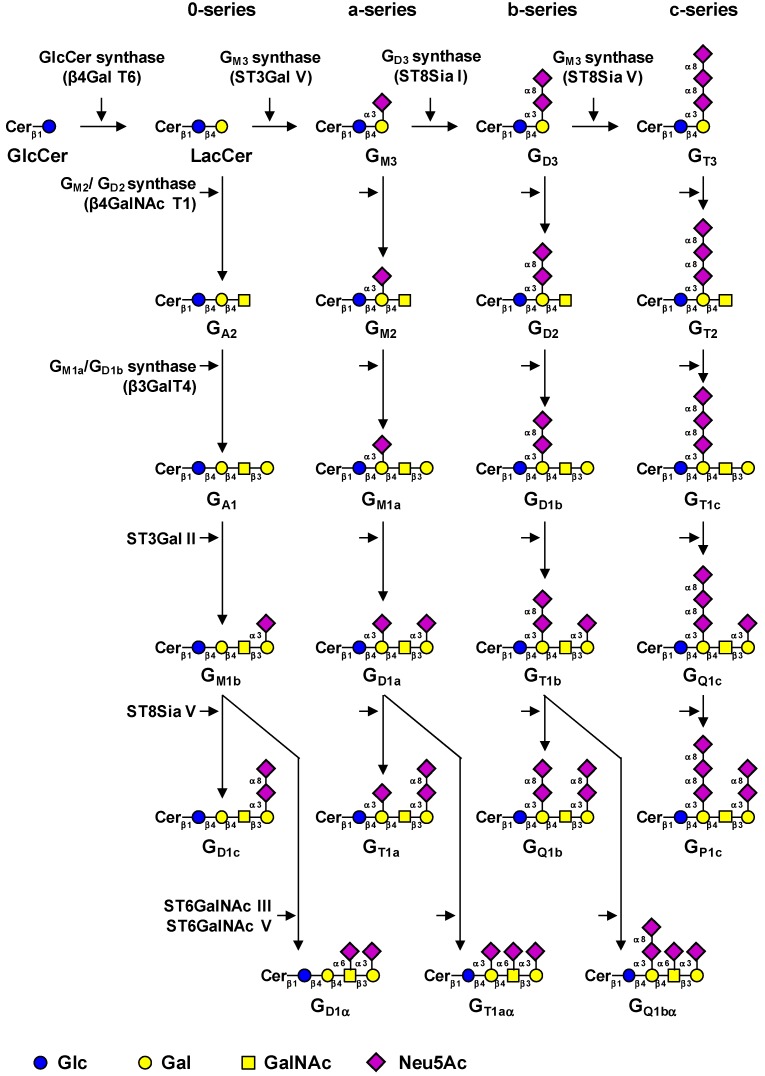
Biosynthesis pathway for gangliosides. Gangliosides are synthesized by the stepwise addition of monosaccharides to ceramide. The sequential action of ST3Gal V (G_M3_ synthase), ST8Sia I (G_D3_ synthase), and ST8Sia V (G_T3_ synthase) leads to the biosythesis of the precursors of a-, b-, and c-series gangliosides, respectively. The 0-series gangliosides are directly synthesized from lactosylceramide. The code names of gangliosides are according to Svennerholm [1].

Gangliosides are glycosphingolipids (GSL) carrying one or several sialic acid residues. According to Svennerholm, gangliosides are classified in four series (0-, a-, b-, and c-series) due to the number of sialic acid residues linked to the lactosylceramide (LacCer) (Figure 1) [1]. Normal human tissues mainly express ‘simple’ gangliosides, from 0- and a-series, whereas ‘complex’ gangliosides from b- and c-series are essentially found in developing tissues, during embryogenesis, and mainly restricted to the nervous system of healthy adults [2]. In humans, the expression of complex gangliosides increases uder pathological conditions including neurodegenerative disorders [3], immune diseases [4], and cacers [5]. For example, G_D3_ and G_D2_ are over-expressed in neuroectoderm-derived tumors such as melanoma, neuroblastoma, and breast cancer, in which they mediate cell proliferation, migration, tumor growth, and angiogenesis [6]. Gangliosides are located on the outer layer of the plasma membrane mainly in glycolipid-enriched microdomains (GEMs), also known as lipid rafts or gangliosides-rich lipid domains. As GEMs are insoluble in detergents at 4 °C, they are also known as detergent-resistant membrane domains. Together with cholesterol, transmembrane proteins, and other glycosphingolipids, gangliosides contribute to the maintenance and dynamic of the membrane organzation. Notably, ganglioside-rich lipid domains are described components of caveolae [7]. 

Quantitative or qualitative (i.e., changes in carbohydrate moiety) modifications of gangliosides can affect GEMs architecture and functions [8]. Amongst the membrane-bound proteins associated to GEMs, many components of signal transduction pathways were identified. The role of GEMs-associated gangliosides in the regulation of signal transduction has been repeatedly reported in a variety of cell lines [9,10,11]. However, the molecular mechanisms sustaining these functions are poorly known. Apprehending the structural heterogeneity and the diversity of interactions between gangliosides and the other components of GEMs should therefore lead to a better understanding of the fine regulation of signal transduction. This has been eased by recent advances in structural analysis of GEMs glycolipids and by the identification of GEMs associated molecules, as reviewed herein. 

## 2. Biosynthesis of Gangliosides

The first step of the biosynthesis of gangliosides is the transfer of a glucose residue onto ceramide (Cer) by the UDP-Glc: ceramide β-glucosyltransferase (GlcCer synthase) encoded by the *UGCG* gene (Table 1) [12]. The next step is the conversion of the glucosylceramide (GlcCer) into lactosylceramide (LacCer), the precursor of the five series of GSL, by the UDP-Gal: GlcCer β1,4-galactosyltransferase (LacCer synthase) [13,14]. The transfer of sialic acid residue to LacCer is then catalyzed by the specific sialyltransferases ST3Gal V (G_M3_ synthase), ST8Sia I (G_D3_ synthase) and ST8Sia V (G_T3_ synthase), all being highly specific for glycolipid substrates [15]. LacCer is the only known substrate for ST3Gal V activity [16] and a loss-of-function mutation in *ST3GAL5* gene is associated with the infantile-onset symptomatic epilepsy syndrome [17]. The G_D3_ synthase ST8Sia I is highly specific for G_M3_ as acceptor substrate [18]. However, the human enzyme was also shown to resialylate its own product G_D3_ creating a chain of 3 (G_T3_), 4 (G_Q3_), or 5 (G_P3_) sialic acid residues, G_Q3_ and G_P3_ being unusual structures recently described [19,20]. The human ST8Sia V exhibits a broader activity toward gangliosides, using G_D3_, but also G_M1b_, G_D1a_ or G_T1b_ as acceptors [21]. LacCer, G_M3_, G_D3_, and G_T3_ are the precursors for 0-, a-, b-, and c-series gangliosides, respectively (Figure 1). Further, monosaccharides can be transferred in a stepwise manner by the β1,4-N-acetyl-galactosaminyltransferase I (G_M2_/G_D2_ synthase) [22] and the β1,3-galactosyltransferase IV (G_M1a_/G_D1b_ synthase) [23], both acting on the four series of gangliosides [24,25]. The terminal Gal residue of the Galβ1-3GalNAc disaccharide can be further sialylated by ST3Gal II [26,27] and ST8Sia V [21], and the GalNAc residue can be sialylated in α2,6-linkage by the sialyltransferases ST6GalNAc III [28] or V [29] to form α-gangliosides (Figure 1). 

**Table 1:** Glycosyltransferases involved in gangliosides biosynthesis. R = LacCer, G_M3_, G_D3_, or G_T3_.

*Gene*	*Common name*	*Main acceptors*	*Accession #*	*Ref.*
UGCG	GlcCer synthase	Ceramide	NM_003358	(12)
B4GALT6	LacCer synthase	Glucosylceramide	NM_004775	(13, 14)
ST3GAL5	G_M3_ synthase	Lactosylceramide	NM_003896	(16)
ST8SIA1	G_D3_ synthase	G_M3_, G_D3_	NM_003034.2	(18)
ST8SIA5	G_T3_ synthase	G_D3_, G_M1b_, G_D1a_, G_T1b_	NM_013305	(21)
B4GALNACT1	G_M2_/G_D2_ synthase	G_A3_, G_M3_, G_D3_, G_T3_	NM_001478.2	(22)
B3GALT4	G_M1a_/G_D1b_ synthase	G_A2_, G_M2_, G_D2_, G_T2_	NM_003782.3	(23)
ST3GAL2	ST3Gal II	Galβ1-3GalNAc-R	NM_006927	(26, 27)
ST6GALNAC3	ST6GalNAc III	Neu5Acα2-3Galβ1-3GalNAc-R	NM_152996	(28)
ST6GALNAC5	ST6GalNAc V	Neu5Acα2-3Galβ1-3GalNAc-R	NM_030965.1	(29)

The first steps of gangliosides synthesis take place in the cis/median-Golgi and the later steps in the trans-Golgi and trans-Golgi network [30]. The regulation of glycosyltransferases (GT) activity is mainly achieved at the transcriptional level [31] and GT genes expression is highly tissue-specific. For example, human *B4GALNACT1* gene is essentially expressed in embryonic tissue and in adult brain, lung and testis. By contrast, *ST3GAL5* is ubiquitously expressed in human tissues [16,32,33]. GT involved in the synthesis of gangliosides can be also regulated by post translational modifications such as *N*-glycosylation, phosphorylation, and dephosphorylation. For example, protein kinases PKA and PKC can activate the G_M2_/G_D2_ synthase while inhibiting the activity of ST3Gal II or G_M1a_/G_D1b_ synthase [34,35,36].

## 3. Regulation of RTKs Signaling by Gangliosides

**Figure 2:**
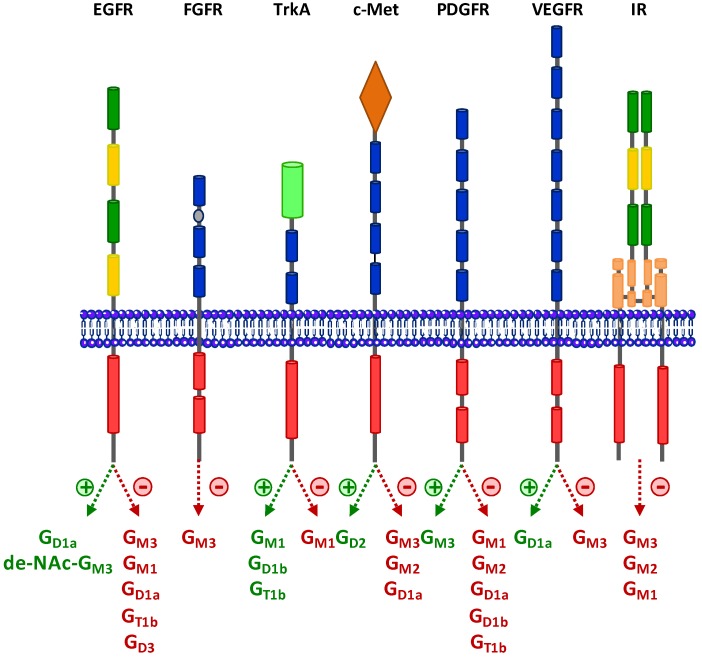
Regulation of RTKs activation by gangliosides. RTKs common structure consists in an extracellular domain containing the ligand binding site, a unique transmembrane domain and a cytoplasmic region containing the tyrosine kinase activity (in red). RTKs are activated by the binding of the ligand that induces receptor dimerization and the autophosphorylation of the intracellular domain. Gangliosides can either inhibit (red arrows) or activate (green arrows) of RTKs signaling, depending on gangliosides expression pattern, cell type, and experimental conditions. Three different mechanisms can be involved: ganglioside/ligand interactions, such as FGF/G_M1_ interaction, the regulation of receptor demerization as for G_M3_ with EGFR, or the regulation of RTKs activity due to the localization inside GEMs as the case for G_M3_ with Insulin receptor (IR). Adapted and updated from [7,37].

Receptor tyrosine kinases (RTKs) are key proteins involved in the control of cellular processes such as survival, proliferation, differentiation, migration and invasion. Fifty-eight RTKs have been identified in Humans. They all share a similar structural organization comprising of an extracellular domain containing the ligand-binding site, a unique transmembrane domain, and a cytoplasmic region containing the tyrosine kinase activity [37]. Usually, RTKs are activated by the binding of the ligand that induces receptor dimerization and the autophosphorylation of the intracellular domain. The role of gangliosides as modulators of signal transduction was first analyzed in the 80’ by the addition of exogenous gangliosides in the medium of cultured cells [38]. However, this approach was rather limited by the unavailability of some specific gangliosides and because it not only modifies the gangliosides pattern but also increases the total amount of cell-membrane-associated gangliosides that can result in non-physiological responses [39]. From 2000, with the progress in the identification of gangliosides biosynthetic enzymes, an increasing number of papers have reported ectopic expression or antisense inhibition strategies targeting specific GT to finely analyze the role of specific gangliosides without modifying the total amount of GSLs. These different approaches have clearly demonstrated that gangliosides are fine regulators of RTKs signaling and that physio-pathological changes in cell membrane ganglioside composition result in different cellular responses [40,41] (Figure 2). 

A number of growth factor receptors, including receptors for epidermal growth factor (EGF), fibroblast growth factor (FGF), platelet-derived growth factor (PDGF), nerve growth factor (NGF), hepatocyte growth factor (HGF), and insulin, were demonstrated to be regulated by gangliosides. RTKs are localized in GEMs with other lipid rafts associated proteins including integrins, tetraspanins, or plexins. Within lipid rafts, RTKs signaling can be negatively or positively regulated by gangliosides by either direct or indirect interactions [7,42]. Changes in gangliosides modify the molecular composition and the structure of glycolipid-enriched microdomains, leading to the reorganization and/or the exclsion of RTKs from GEMs [43,44,45]. Finally, it was also demonstrated that the crosstalk between RTKs subunits and other lipid rafts associated proteins is also regulated by gangliosides. 

### 3.1. Epidermal Growth Factor Receptor (EGFR)

Several studies have shown that G_M3_ is able to bind to the extracellular domain and inhibit the kinase activity of EGFR in a variety of cell lines. The effect of gangliosides on EGF-dependent tyrosine phosphorylation of EGFR was first demonstrated in human epidermoid carcinoma cell line A431 [38]. G_M3_ added exogenously to cells in culture was shown to inhibit EGFR autophosphorylation [38,46] whereas de-N-acetyl-G_M3_ (II_3_NeuNH_2_LacCer) enhances serine phosphorylation independently of receptor-receptor interaction [47,48]. Similarly, depletion of G_M3_ in A431 cells by PDMP (d-threo-1-phenyl-2-decannoylamino-3-morpholino-1-propanol), which inhibits the GlcCer synthase, increased EGFR autophosphorylation upon EGF stimulation [49]. G_M3_ directly interacts with EGFR on a site distinct from the EGF-binding site [50] through direct carbohydrate-carbohydrate interactions between G_M3_ and terminal GlcNAc residues on EGFR *N*-glycans [51,52]. G_M3_ binding to EGFR is enhanced after glycosidase-treatment that exposes *N*-glycan terminal GlcNAc, whereas G_M3_ does not bind to EGFR from ManIB-knocked down cells that accumulates high mannose-type (i.e., immature form lacking terminal GlcNAc) *N*-glycans [52]. This was further confirmed using UDP-Gal 4-epimerase defective ldlD cells transfected with EGFR gene, in which high amount of terminal GlcNAc residues (that accumulate due to the lack of UDP-Gal) is correlated with an inhibitory effect of G_M3_ on EGFR [53]. G_M3_ was also shown to suppress murine hepatoma cell motility by inhibiting EGFR phosphorylation and the downstream PI3K/Akt signaling pathway [54]. 

More recently, it has been reported that G_M3_ and the tetraspanin tumor suppressor CD82 induce synergistic inhibition of migration Hepa1-6 cells by reducing EGFR phosphorylation [55]. By reconstituting human EGFR into proteoliposomes, it was shown that G_M3_ inhibits the structural transition from inactive EGFR to signaling EGFR dimer, by preventing the autophosphorylation of the intracellular kinase domain in response to ligand binding [56]. In parallel, stable transfection of the G_D3_ synthase in CHO-K1 cells induces cell surface expression of G_D3_ and decreases EGFR phosphorlation and Erk2 activation upon EGF stimulation [57]. Inhibition of EGFR phosphorylation and cell proliferation due to G_M3_, G_M1_, G_D1a_, and G_T1b_ treatment were also reported in human neuroblastoma cells [58]. In normal human dermal fibroblasts, G_D1a_ promotes the ligand-independent EGFR dimerization and enhances EGFR-mediated activation of the mitogen-activated protein kinase (MAPK) signaling pathway [59]. Accordingly, it was also shown that EGFR phosphorylation is signiicantly reduced with the knockdown of ST3Gal II, the enzyme that converts G_M1_ to G_D1a_ [60]. 

### 3.2. Fibroblast Growth Factor Receptor (FGFR)

FGFR participates in many developmental, homeostatic and healing processes including neurogenesis, axon growth, differentiation, and neuronal survival [61]. The negative effect of G_M3_ on FGFR activation and tyrosine phosphorylation was first demonstrated in cultured retinal glial cells [62]. The interaction of G_M3_ with FGFR was hinted by confocal microscopy analysis in human lung embryonic fibroblast WI38, showing co-localization of G_M3_ and FGFR in the GEM fraction [63]. Moreover, G_M3_ depletion by GlcCer synthase inhibition enhances tyrosine phosphorylation of FGFR, activates PI3K/Akt pathway and increases the interactions of FGFR with α3/α5/β1 integrins [64]. This demonstrated that integrin-FGFR cross-talk is regulated by G_M3_ within the ganglioside-enriched microdomains. 

### 3.3. Neurotrophins Receptors

It has been clearly demonstrated that G_M1_ ganglioside regulates neurotrophins receptors both *in vivo* and in cell cultures [65,66,67]. In rat pheochromocytoma PC12 cells, the addition of exogenous G_M1_ to cell culture enhances NGF/TrkA signaling and protects neuronal cells from serum deprivation-induced apoptosis [65]. On the contrary, the over-expression of G_M1_ by the transfection of β3GalT4 cDNA, the enzyme that converts G_M2_ in G_M1_, inhibited NGF-induced TrkA dimerization and phosphorylation as well as the downstream pathway [68]. According to the authors, this opposite effect of G_M1_ in PC12 was due to the high concentration of G_M1_ at the plasma membrane in β3GalT4 expressing cells that modulated membrane fluidity, impeding the NGF receptor localization within the lipid rafts [68]. In parallel, the introduction of the G_D3_ synthase gene into PC12 cells resulted in the over-expression of G_D1b_ and G_T1b_. These gangliosides triggered a conformational change of TrkA that formed a constitutively active dimer, activating its downstream signal pathways, including Erk1/2 and PI3K/Akt, and leading to a marked enhancement of cell proliferation [69,70].

### 3.4. Hepatocyte Growth Factor Receptor c-Met

**Figure 3:**
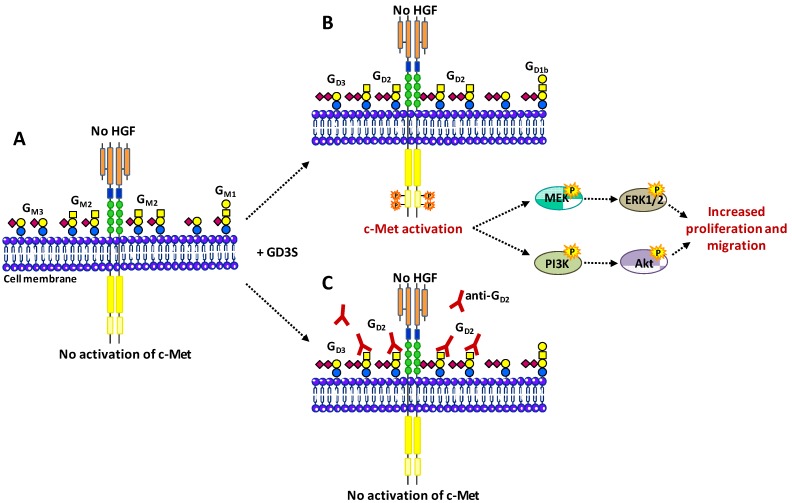
Activation of c-Met by G_D2_ ganglioside. (**A**) MDA-MB-231 breast cancer cells express mainly G_M3_ and G_M2_. (**B**) The expression of the G_D3_ synthase induces the accumlation of b- and c-series gangliosides, mainly G_D2_. This leads to the activation of c-Met in the absence of HGF and increases proliferation and migration through PI3K/Akt and MEK/Erk pathways. (**C**) Anti-G_D2_ mAb used in competition assays inhibits c-Met phosphorylation and cell proliferation [72,74].

In HCV29 bladder epithelial cells, motility and growth are modulated by the expression of a-series gangliosides. In the presence of Ca^2+^, G_M3_, and G_M2_ form heterodimers that specifically interact with tetraspanin CD82, thus impairing the trans-phosphorylation of c-Met receptor, the recruiting of Grb2 and the activation of PI3K/Akt and MEK/Erk pathways [44,71]. Similarly, the ganglioside-dependent activation of c-Met receptor was also recently demonstrated in breast cancer cells [72]. The expression of the G_D3_ synthase in MDA-MB-231 breast cancer cells induced the cell surface accumulation of b- and c- series gangliosides including G_D3_, G_D2_, and G_T3_ [73,74]. Of these complex gangliosides, G_D2_ was found to be involved in the activation of c-Met, and the subsequent activation of MEK/Erk and PI3K/Akt signaling pathways, leading to enhanced cell migration and proliferation. This was shown by competition assays using anti-G_D2_ mAb that inhibited c-Met phosphorylation (Figure 3), demonstrating the role of the G_D2_ glycan moiety in c-Met activation [74]. Moreover, silencing of the G_M2_/G_D2_ synthase (β4GalNAc T1) efficiently reduced both G_D2_ expression and c-Met phosphorylation. Of importance, the G_D2_-dependent activation of c-Met occurred in the absence of HGF [72]. On the other hand, the ganglioside G_D1a_ that belongs to the a-series, was shown to inhibit HGF-induced motility and scattering of mouse osteosarcoma cell variant FBJ-LL cells through the suppression of phosphorylation of c-Met [75]. 

### 3.5. Platelet-Derived Growth Factor Receptor (PDGFR)

Various gangliosides were shown to inhibit PDGF-dependent tyrosine phosphorylation of PDGFR in several cell types including Swiss 3T3 [76], human glioma cells [77], and neuroblastoma SH-SY5Y cells [78]. Of the tested gangliosides (G_M1_, G_M2_, G_M3, _G_D1a_, G_D1b_, G_D3_, and G_T1b_), only G_M3_ did not inhibit the dimerization of PDGFR [79] but could facilitate PDGF-dependent receptor activation, as an anti-G_M3_ antibody was found to inhibit PDGF receptor activation in T51B liver epithelial cells [80]. Amongst the gangliosides inhibiting PDGFR, G_M1_ was the most studied. In human glioma cells, G_M1_ treatment resulted in reduced phosphorylation of specific tyrosine residues of the cytoplasmic tail of PDGFR [81]. However, it was later shown that the cytoplasmic domain of PDGFR was not required for G_M1_-dependent inhibition of the receptor [82]. Indeed, G_M1_ inhibition of PDGFR seems to be rather due to the exclusion of the receptor from glycolipid-enriched microdomains [83]. Recently, it was shown that the Csk binding protein PAG (Phosphoprotein Associated with Glycosphingolipid-enriched micro-domains) [84] regulates PDGFR partitioning in caveolae and its association with SRC family protein tyrosine kinases (SFK) by controlling G_M1_ levels at the plasma membrane [85]. 

### 3.6. Vascular Endothelial Growth Factor Receptor (VEGFR)

Several pieces of evidence have suggested that gangliosides also modulate tumor angiogenesis by controlling the activation of VEGF receptors FLT1 (VEGFR-1) and FLK1/KDR (VEGFR-2). It has been shown that ganglioside enrichment in human umbilical vein vascular endothelial cells (HUVEC) induces VEGFR dimerization and autophosphorylation at very low VEGF concentrations [86] and icubation of HUVEC with exogenous G_D1a_ increases VEGF-induced proliferation and migration [87]. G_M3_ is implicated in the decrease of VEGFR-2 phosphorylation and subsequent inhibition of Akt downstream signaling pathway in HUVECs [88,89]. It was also shown that G_M3_ decreases VEGF-induced VEGFR-2 activation by blocking receptor dimerization and the binding of VEGF to VEGFR-2 through a G_M3_-specific interaction with the extracellular domain of VEGFR-2 [90]. In contrast, the elevation of the proportion of G_M3_ in CT-2A malignant mouse astrocytoma cells using G_M2_/G_D2_ synthase shRNA reduces tumor-induced angiogenesis [91]. Moreover, the antisense inhibition of β3GalT4 expression in the highly angiogenic CT-2A astrocytoma cells, which mainly express G_D1a_, increases G_M3_ content while reducing G_D1a_ and reduces growth, VEGF gene and protein expression, and vascularity [88]. Finally, it has been recently shown using a mass spectrometry-based approach that the soluble form of VEGFR-1 (sFLT1) binds to G_M3_ in lipid rafts on the surface of podocytes (kidney glomerular pericytes), promoting adhesion and rapid actin reorganization [92,93].

### 3.7. Insulin Receptor

G_M3_ has been described as a negative regulator of insulin signaling, partially responsible for insulin resistance. In 3T3-L1 adipocytes, insulin resistance induced by tumor necrosis factor (TNF) is accompanied by an increased expression of G_M3_ synthase activity and G_M3_ ganglioside [94]. The increased interaction between insulin receptor and G_M3_ leads to the dissociation of insulin receptor (IR) from caveolae [95]. Moreover, inhibition of ganglioside biosynthesis by PDMP, a specific inhibitor of the GlcCer synthase, restores insulin signaling, whereas addition of exogenous G_M3_ inhibits the IR substrate 1 (IRS-1) phosphorylation and IR signaling pathway [94,96]. Similar results were obtained with G_M3_ synthase mutant mice that show an enhanced IR phosphorylation and a heightened sensitivity to insulin [97]. In parallel, hepatic over-expression of the membrane-associated sialidase NEU3 in C57BL/6 mice reduces G_M3 _level in the liver, improving insulin sensitivity [98]. It was also demonstrated that G_M3_ interacts with a lysine residue of IR beta-subunit localized above the transmembrane domain and induces the dissociation of the IR-caveolin-1 complex, which is essential for insulin signaling [99]. Finally, G_M1_ and G_M2_ were also shown to inhibit IR phosphorylation in *in vitro* assay [100].

## 4. Conclusion

To conclude, it is now clear that gangliosides regulate RTKs within glycolipid-enriched microdomains either by inhibiting the dimerization and autophosphorylation of the receptors induced by specific ligands, or activating receptors signaling without ligand binding. Moreover, the activation or inhibition of RTKs is dependent on the glycan structure of gangliosides and cellular context. From a general point of view, monosialogangliosides, such as G_M3_ or G_M1_ can be considered as negative regulators of RTKs signaling whereas disialogangliosides including G_D2_, G_D1a_, or G_D1b_ mostly activated RTKs-mediated signal transduction. However, the molecular mechanisms by which gangliosides regulate RTKs remain poorly understood. Direct interactions between carbohydrate moiety of gangliosides and RTKs have been clearly identified as demonstrated for G_M3_ inhibition of EGFR, but direct carbohydrate-carbohydrate interactions cannot explain the different observed effects. Gangliosides regulation of RTKs also involved the reorganization of GEMs due to the change in ganglioside composition that induces the dissociation of RTKs from glycolipid-enriched microdomains, resulting in a reduced phosphorylation of the receptors as it has been demonstrated for insulin receptor. Indirect interactions with other GEMs associated transmembrane proteins including integrins and tetraspanins, can also be involved in the regulation of RTKs by gangliosides, as it has been demostrated for c-Met receptor. In parallel, the regulation of RTKs by gangliosides is highly depending on the carbohydrate moiety of gangliosides as shown for c-Met receptor, which is activated by G_D2_ whereas G_D3_ has no effect. The fine recognition of the glycan part of gangliosides should involve membrane lectin domains, able to discriminate between subtle changes in ganglioside glycans. The use of emergent technologies such as glycan arrays and photocrosslinking should enable to identification of such lectin domains [101,102]. Finally, changes in ganglioside composition occur in pathological conditions and are observed in a variety of cancers, mainly in neuro-ectoderm-related cancers. The understanding of the mechanisms by which gangliosides modify RTKs signaling is therefore of first importance to identify new targets in cancer therapy.
